# Enhanced Electrocatalysis on Copper Nanostructures: Role of the Oxidation State in Sulfite Oxidation

**DOI:** 10.1021/acscatal.3c05897

**Published:** 2024-07-19

**Authors:** Esperanza Fernández-García, Pablo Merino, Nerea González-Rodríguez, Lidia Martínez, María del Pozo, Javier Prieto, Elías Blanco, Gonzalo Santoro, Carmen Quintana, María Dolores Petit-Domínguez, Elena Casero, Luis Vázquez, José I. Martínez, José A. Martín-Gago

**Affiliations:** † Departamento de Química Analítica y Análisis Instrumental, Facultad de Ciencias, c/Francisco Tomás y Valiente, Campus de Excelencia de la Universidad Autónoma de Madrid, Madrid 28049, Spain; ‡ Instituto de Ciencia de Materiales de Madrid ICMM (CSIC), Madrid E-28049, Spain; § Instituto de Estructura de la Materia (IEM), CSIC, c/Serrano 121, Madrid 28006, Spain

**Keywords:** electrooxidation, copper oxidation state, DFT calculations, thermochemistry, sulfite, XPS, TEM, AFM

## Abstract

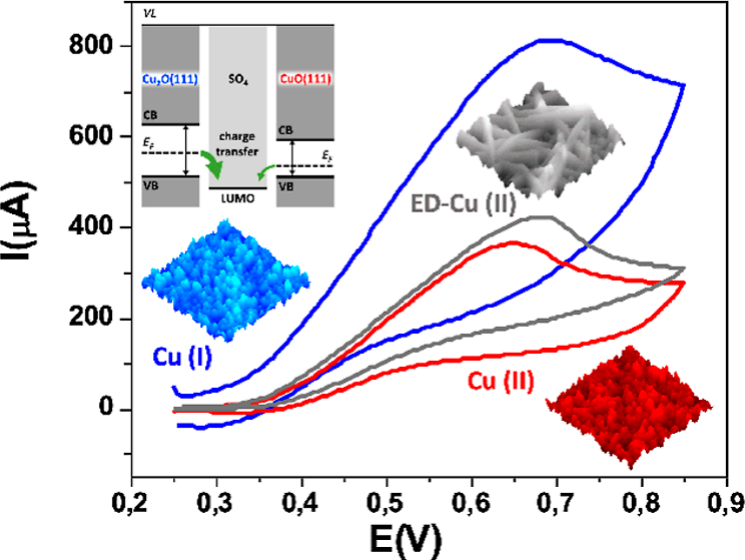

The influence of surface morphology and the oxidation state on the electrocatalytic activity of nanostructured electrodes is well recognized, yet disentangling their individual roles in specific reactions remains challenging. Here, we investigated the electrooxidation of sulfite ions in an alkaline environment using cyclic voltammetry on copper oxide nanostructured electrodes with different oxidation states and morphologies but with similar active areas. To this aim, we synthesized nanostructured Cu films made of nanoparticles or nanorods on top of glassy carbon electrodes. Our findings showed an enhanced sensitivity and a lower detection threshold when utilizing Cu(I) over Cu(II). Density functional theory-based thermochemical analysis revealed the underlying oxidation mechanism, indicating that while the energy gain associated with the process is comparable for both oxide surfaces, the desorption energy barrier for the resulting sulfate molecules is three times higher on Cu(II). This becomes the limiting step of the reaction kinetics and diminishes the overall electrooxidation efficiency. Our proposed mechanism relies on the tautomerization of hydroxyl groups confined on the surface of Cu-based electrodes. This mechanism might be applicable to electrochemical reactions involving other sulfur compounds that hold technological significance.

## Introduction

Copper oxide nanoparticles (CuNPs), specifically CuO and Cu_2_O, have emerged as crucial catalysts in diverse scientific areas, heralding advances in green chemistry and sustainable energy. Their distinctive physicochemical characteristics, such as a high surface-to-volume ratio, earth abundance, cost effectiveness, and chemical and electrochemical robustness, render them exceptionally effective across an array of catalytic reactions.^1−4^ CuNPs have been instrumental in steering hydrocarbon selectivity in catalytic electroreduction of CO_2_,^5,6^ creating antibacterial coatings,^2,7^ designing electrochemical sensors,^8−10^ and selective hydrogenating alkynes and alkadienes,^11^ among other applications.

For electrochemical sensing, both CuO and Cu_2_O nanoparticles are extensively used because of their good electrochemical activity and their ability to promote electron transfer reactions.^12^ Moreover, the size and shape tunability of these nanoparticles further allows for the optimization of their catalytic performance.^6,13−15^

Although the role of structural properties of metal NPs on the electrocatalytic performance has been already studied,^16,17^ the impact of copper’s oxidation state has not been fully explored due, among other things, to the difficulty in isolating their contribution to the catalytic activity. However, given that specific electronic states are inherently involved in catalytic reactions, the oxidation state of copper shall be a determining factor of the efficiency. To address this fundamental issue, we have synthesized on a glassy carbon electrode (GCE) Cu films formed by nanoparticles or nanorods, exhibiting different oxidation states. By maintaining similar morphologies across different copper films while keeping the same active area, we thus isolate the effect of the oxidation state as a variable and demonstrate its dominant role in sulfite oxidation.

To assess the efficiency of the different Cu nanostructures, we have chosen a model system, sulfur-containing compounds. Sulfite detection and removal is of great importance for environmental protection, industrial processes, food preservatives, pharmaceuticals, and quality control because of their potential toxicity.^18^ Its electrochemical oxidation to sulfate is the base of many amperometric sensors and a paradigmatic model system in electrochemistry. Although their relevance is significant, a mechanism for their catalytic oxidation has not been proposed yet. Density functional theory (DFT) calculations have recently shown their capability to obtain atomistic insights into several processes involving nitrogenated species,^19^ but they have not yet been applied to unveil catalytic oxidation mechanisms of sulfur-containing compounds.

Disentangling the structural characteristics from the oxidation states of the catalysts in a reaction presents a complex yet crucial challenge in catalysis research.^16^ Recent advancements in in-operando techniques have significantly contributed to our understanding of catalytic reactions in real time, offering a window into the dynamic changes during a reaction.^20,21^ However, in our current investigation, we have thoroughly assessed the stability of our nanomaterials prior to and following our experiments. Our findings are based on the premise that the inherent stability of the nanomaterials used makes unnecessary real-time analysis as the static nature of the catalysts under our experimental conditions suggests minimal dynamic changes in the structure or oxidation states.

Here, we present an oxidation mechanism for sulfite on both the Cu(I) and Cu(II) surfaces. Our proposed mechanism confirms our experimental data from voltammetric measurements, indicating superior electrocatalytic activity on Cu(I) electrodes. A pivotal aspect of this mechanism involves a low-barrier tautomerization step where the hydrogen atom of a hydroxyl moiety, trapped by the sulfite anion, transitions to another hydroxyl, releasing a water molecule in the process. While the reaction proceeds with a favorable energy gain on both catalytic surfaces, the difference in performance arises from the higher adsorption energy of the formed sulfate on Cu(II). This increased energy leads to a larger desorption barrier when transitioning from the electrode to the electrolyte. This study unveils a viable atomistic mechanism of sulfite oxidation and may permit the design of better copper-based electrodes for catalysis.

## Results and Discussion

### X-Ray Photoelectron Spectroscopy Characterization of the Cu-Based Nanostructured Electrodes

We have synthesized four distinct copper-based nanostructured films on a GCE (see Sections 1.1 and 1.2 in the Supporting Information). On one hand, we have fabricated three different electrodes based on the deposition of copper NPs synthesized in the gas phase [Cu^0^, Cu_2_O, and CuO nanoparticles, hereafter denoted as GP-Cu^0^, GP-Cu(I), and GP-Cu(II), respectively]. The main advantages of gas phase synthesis are the very narrow NP size distribution, absence of contaminants, and precise control of the chemical state by varying the oxygen concentration during the process.^5,22^ On the other hand, we have synthesized an electrodeposited Cu film (ED-Cu) exhibiting a nanorod morphology. These films were prepared following a protocol consisting in the electrochemical reduction of a copper salt resulting in a Cu film onto the GCE, which is later subjected to cyclic voltammetric scans in an alkaline medium,^23,24^ see Section 1.2 in the Supporting Information and Figures S1 and S2 for more details about the protocol.

**Figure 1:**
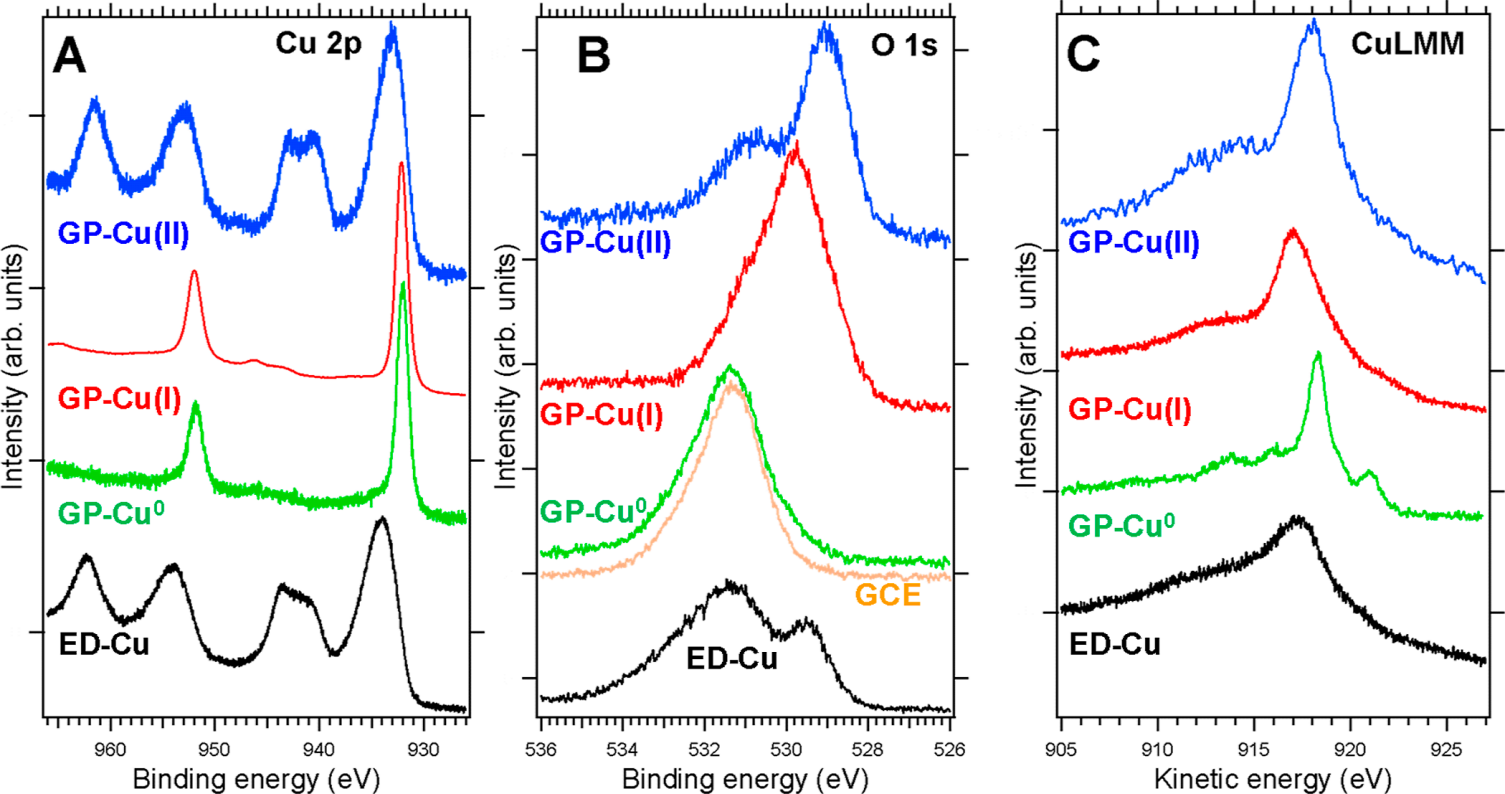
XPS spectra of the different Cu-based nanostructured films. (A) Cu 2p and (B) O 1s core levels and (C) Cu LMM Auger spectra of GP-Cu^0^ (green), GP-Cu(I) (red), GP-Cu(II) (blue) nanoparticles, and ED-Cu (black) nanorods. Spectra from gas phase synthesized nanoparticles were acquired in situ after growth without exposing the nanoparticles to ambient conditions. The orange line in (B) corresponds to the O 1s spectrum of the GCE surface. The spectra were vertically shifted for clarity.

The oxidation states of these four samples have been determined by X-ray photoelectron spectroscopy (XPS). Figure 1 shows representative Cu 2p, O 1s, and Cu LMM spectra of the gas phase synthesized Cu-based nanoparticles, namely, GP-Cu(II), GP-Cu(I), and GP-Cu^0^, respectively, measured in situ, as well as the Cu-based nanorods (ED-Cu), measured ex situ, all on glassy carbon. The Cu 2p core level blue spectrum of Figure 1A (GP-Cu(II)) can be assigned to a +2 oxidation state, from both the binding energy (BE) of the Cu 2p_3/2_ (932.9 eV) and the pronounced shakeup satellites emerging at 940.6, 943.0, and 961.6 eV.^5,22^ In the Cu 2p red spectrum [GP-Cu(I)], the Cu 2p_3/2_ peak is shifted to 931.9 eV.^25,26^ Such a feature is indicative of a +1 oxidation state of the NPs. In the absence of an O_2_ flow during NP growth (green Cu 2p spectrum, GP-Cu^0^), the Cu 2p_3/2_ peak is located at 932.0 eV and no shakeup satellites are observed, in agreement with a metallic character of the NPs, i.e., GP-Cu^0^. The Cu 2p black spectrum corresponding to ED-Cu shows clear shakeup satellites that are a fingerprint of the presence of Cu(II) that could be ascribed to CuO. In addition, in this case, several features indicate the presence of hydroxides,^25^ namely, the Cu 2p_3/2_ peak appearing at a BE of 934.0 eV, the shape of the Cu 2p shakeup satellites, and their shift to higher energies with respect to that of GP-Cu(II), as well as the existence of the Cu 2p shakeup satellites at lower energy, around 943 eV.

The oxidation state of the gas phase and electrochemically synthesized Cu-based structures can also be traced in the O 1s spectra (Figure 1B). The O 1s peaks of GP-Cu(II) and GP-Cu(I) have their maxima at 529.1 and 529.7 eV, respectively. The contribution at 531.1 eV can be attributed to adventitious oxygen coming from the glassy carbon substrate from the comparison with the orange spectrum. The interpretation of the O 1s peak of the ED-Cu black spectrum is more complicated. The peak at around 532.4 eV can be ascribed to H_2_O-related products on the whole sample as a result of the sample immersion in aqueous solutions during the treatment. The low energy shoulder at 529.5 eV is due to Cu(II).

The different oxidation states of Cu in GP-Cu^0^, GP-Cu(I), GP-Cu(II), and ED-Cu nanostructured electrodes are also reflected in the Auger Cu LMM spectra (Figure 1C). The Cu LMM Auger maxima of GP-Cu(II), GP-Cu(I), and GP-Cu^0^ (blue, red, and green spectra, respectively) appear at kinetic energies (KEs) of 918.1, 916.9, and 918.6 eV, respectively. Such energy shifts are characteristic for the Auger spectra of CuO, Cu_2_O, and metallic Cu compounds and are a hallmark of the chemical state of Cu nanostructures in the three different oxidation states. The ED-Cu sample shows the Cu LMM Auger at a KE of 917.4 eV, suggesting a mixture of Cu(OH)_2_ with CuO.

### Transmission Electron Microscopy and Atomic Force Microscopy Characterization of the Cu-Based Nanostructured Electrodes

**Figure 2:**
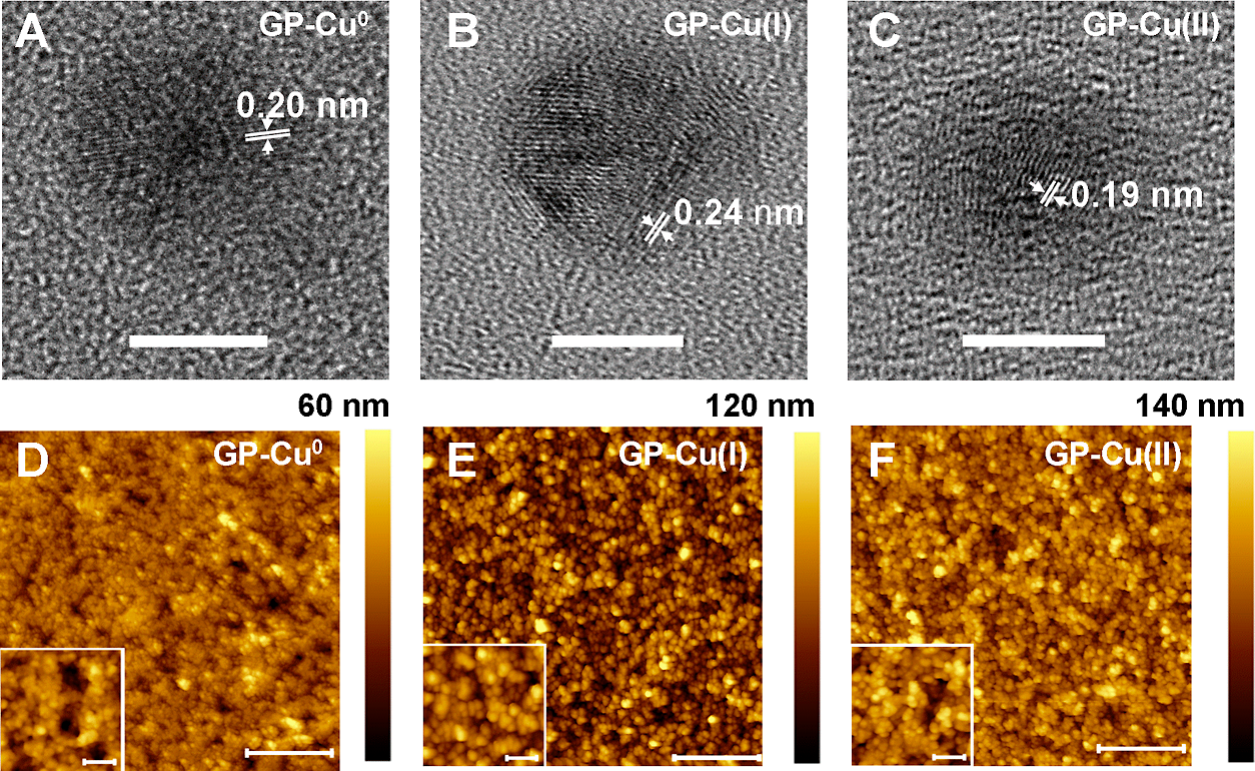
TEM/AFM images of (A/D) GP-Cu^0^, (B/E) GP-Cu(I), and (C/F) GP-Cu(II). The scale bars in the TEM and AFM images correspond to 5 and 500 nm, respectively. AFM insets: Zoomed-in views of the different morphologies. The scale bars correspond to 100 nm.

To evaluate the NP size in the GP-Cu^0^, GP-Cu(I), and GP-Cu(II) electrodes, we prepared low coverage NP deposits on transmission electron microscopy (TEM) grids (Figure S3). The average NP diameters extracted from the TEM analysis are 7.2 ± 0.6, 8.7 ± 0.7, and 7 ± 1 nm for GP-Cu^0^, GP-Cu(I), and GP-Cu(II), respectively. Thus, the NPs are of similar size. Figure 2A–C shows high-magnification TEM images of individual GP-NPs, revealing a crystalline structure, irrespective of their oxidation state. The corresponding interplanar distances of the NPs are 0.20, 0.24, and 0.19 nm, respectively, which correspond to the lattice spacings of (111) Cu,^27^ (111) Cu_2_O,^28^ and (110) CuO,^29^ respectively, in agreement with the stoichiometry derived from the XPS measurements for GP-Cu^0^, GP-Cu(I), and GP-Cu(II).

Figure 2D–F shows representative atomic force microscopy (AFM) images of GP-Cu^0^, GP-Cu(I), and GP-Cu(II) nanostructured electrodes, respectively. In all cases, the morphology consists in a multilayer film of NPs. As the deposited film covers the whole surface, it is not possible to measure its thickness. However, an underestimation can be obtained from the measurement of the difference between the lowest and highest locations in the image, which yields a minimum value of 60 nm for the GP-Cu^0^ sample and close to 130 nm for the oxide ones (i.e., at least 9 and 16 layers of NPs, respectively). The corresponding root-mean-square (*rms*) roughness values are 5.0 ± 0.6, 17 ± 2, and 14 ± 2 nm, for the GP-Cu^0^, GP-Cu(I), and GP-Cu(II) samples, respectively. Thus, the GP-Cu(I) and GP-Cu(II) electrodes show a very similar roughness.

We performed a fractal dimensional analysis on the AFM images according to the procedure described in Section 4 of the Supporting Information (Figures S4 and S5). The aim of this study is to characterize the porosity of GP-Cu(I) and GP-Cu(II) (see Figure S6 in the Supporting Information). As shown in Figure S6A, the fractal behavior of both films is analogous since the perimeter/area data of both samples overlap, which indicates that their porous networks are also alike.^30^ Overall, the similar roughness, nanoparticle size, and porous structure of GP-Cu(I) and GP-Cu(II) indicate that both electrodes present very similar morphological characteristics, which is relevant for a proper comparison of the electrocatalytic activity of both electrodes.

**Figure 3:**
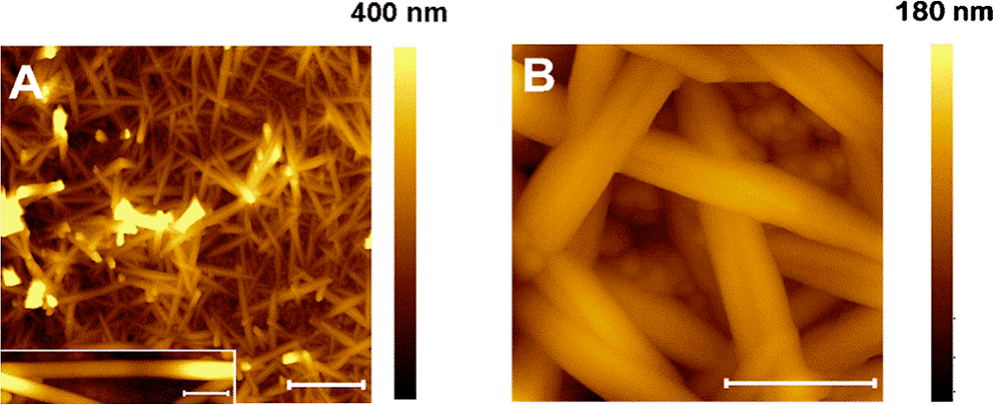
(A) AFM image of the ED-Cu sample. The bar corresponds to 1 μm. Inset: A detail of a single nanorod. The bar indicates 200 nm. (B) Detail of the nanofiber network on the Cu NPs background. The bar indicates 200 nm.

Likewise, the ED-Cu sample was imaged by AFM (Figure 3). The images show a network of nanorods with typical widths of 60–80 nm and lengths of 1.2–1.4 μm. The *rms* roughness value of the ED-Cu electrode is considerably higher (83 ± 9 nm) in comparison with those of the rest of the prepared electrodes. Figure 3B shows a detail of one of these nanorods that displays a very smooth surface with a surface roughness smaller than 1 nm. This figure also reveals the growth of the nanorod network on top of a background layer of NPs.

Table 1 summarizes the main morphological parameters of the four nanostructured electrodes considered in this article. The ED-Cu electrode, contrary to the GP electrodes, differs greatly in terms of roughness due to its nanowire network morphology. Nevertheless, all of the nanostructured electrodes present a very similar active surface area (see Section 2 in the Supporting Information for measurement details). Note that for obtaining the surface area values, five different electrodes for each system were measured. The mean values and their corresponding errors (standard deviations) are given in Table 1. It is worth noting that as the precise quantification of the real surface area becomes particularly complex when electrodes are modified with nanomaterials;^31−33^ the actual errors may be larger. This complexity arises from the absence of a universally accepted methodology for area quantification, which may vary depending on the specific nanomaterial in question and its size (Section 2 in the Supporting Information). Despite these drawbacks, the considerable morphological similarity, in terms of roughness, porosity, and nanoparticle size, between GP-Cu(I) and GP-Cu(II), which are the main focus of this work. Therefore, it is reasonable to assume that their effective surface areas are comparable.

**Table 1:** Morphological Data of the Different Nanostructured Electrodes^a^

	ED-Cu	GP-Cu^0^	GP-Cu(I)	GP-Cu(II)
morphology	nanorod	nanoparticles	nanoparticles	nanoparticles
NP size (nm)		7.2 ± 0.6	8.7 ± 0.7	7 ± 1
film thickness (nm)	∼120	>60	>130	>130
surface roughness (nm)	83 ± 9	5 ± 0.6	17 ± 2	14 ± 2
active surface area (cm^2^)	0.104 ± 0.004		0.105 ± 0.008	0.113 ± 0.007

a These data come from five modified electrodes in each case, being the errors their corresponding standard deviations.

Other possible issue is the eventual evolution of Cu(I) to Cu(II) over long temporal periods. Even though in small nanoparticles, this evolution seems to be retarded,^22^ we can expect a decay in a long-term scale to Cu(II). Notwithstanding this, our electrochemical experiments for sulfite determination are completed within an approximate 1 h window. Within this duration, our data consistently show no notable deviations from the beginning of the experimental procedures. This time frame is critical to ensure that the stoichiometric effects documented in our study accurately reflect the catalyst’s immediate performance. Nevertheless, we have performed emersion experiments, i.e., ex situ surface morphological (Figures S7 and S8) and chemical analysis (Figure S9), of the three samples after the electrocatalytic process. These analyses, detailed in Sections 6 and 7 of the Supporting Information, confirm that the electrocatalytic process induces neither substantial morphological nor chemical alterations on the surfaces of the GP-Cu(I), GP-Cu(II), and ED-Cu electrodes.

### Catalytic Response of Copper-Based GCEs toward Sulfite Oxidation

Sulfite oxidation to sulfate by cyclic voltammetry permits us to address the dependence of the electrode response on the copper oxidation state. First, we confirmed that the observed oxidation peak is exclusively related to sulfite oxidation by comparing the electrochemical response of the three electrodes, namely, GP-Cu(I), GP-Cu(II), and ED-Cu, obtained in 0.01 M NaOH both in the absence and in the presence of 10 mM Na_2_SO_3_ (see Section 8 and Figure S10 in the Supporting Information). Furthermore, we also confirmed the effectiveness of modifying the GCE with Cu nanostructures for sulfite determination by comparing its corresponding voltammogram (Figure S10C, curve 2) to that of the unmodified GCE (Figure S10C, curve 4).

**Figure 4:**
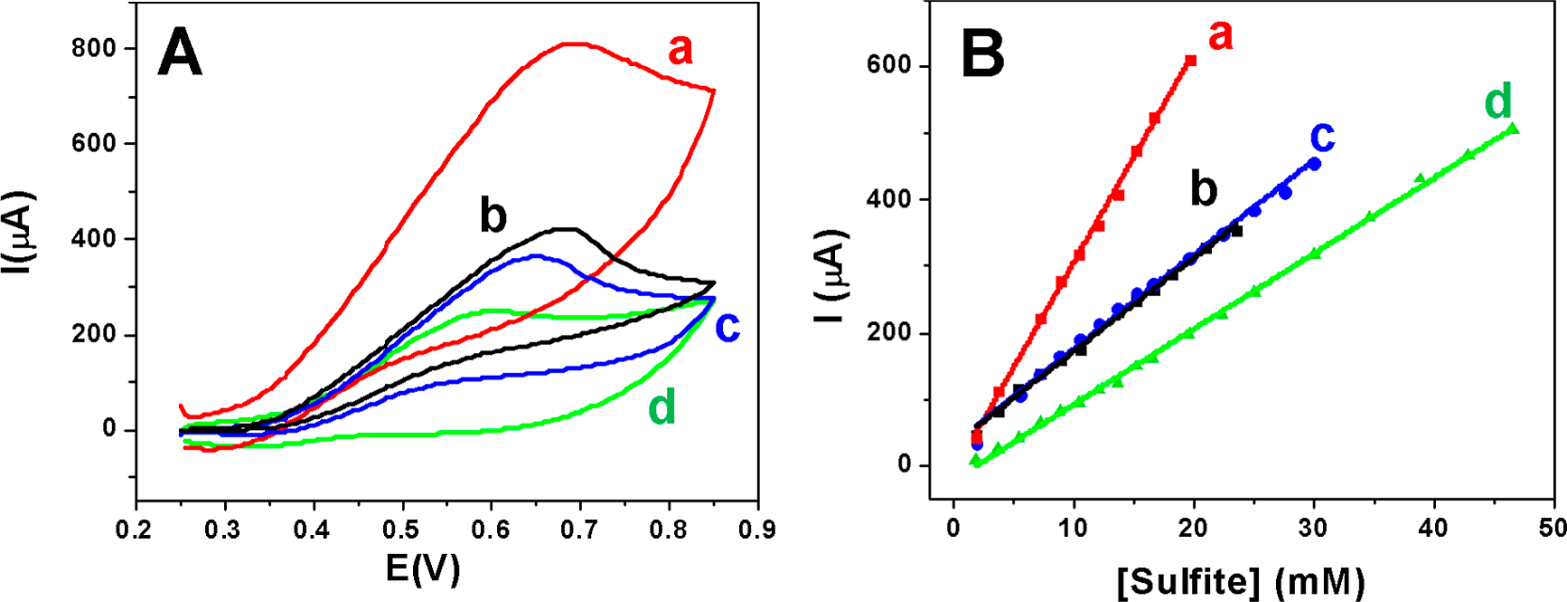
(A) Cyclic voltammograms in a solution containing Na_2_SO_3_ 10 mM and NaOH 0.01 M (scan rate = 50 mV s^–1^) and (B) linear range of calibration curves obtained with [(a) red line] GP-Cu(I), [(b) black line] ED-Cu, [(c) blue line] GP-Cu(II), and [(d) green line] GP-Cu^0^ electrodes. Note that the *y*-axis refers to the current directly measured during the experiments.

We compare in Figure 4 the electrochemical response of the GCE modified with those of ED-Cu (scan b), GP-Cu(I) (scan a), GP-Cu(II) (scan c), and GP-Cu^0^ (scan d).

In all cases, the electrochemical response toward sulfite leads to a well-defined anodic peak about 0.65 V with a high current intensity, which evidences the effectiveness of all Cu-based electrodes for sulfite detection. We find that electrodes based on Cu(II), either ED-Cu or GP-Cu(II), yield electrochemical signals about two times higher than that obtained on GP-Cu^0^. Likewise, the GP-Cu(I) electrode displays an electrochemical signal about three and half times higher than that of GP-Cu^0^. Figure 4B presents the linear concentration ranges of the calibration curves obtained from the cyclic voltammetric responses of the four different modified electrodes toward increasing sulfite concentrations. From the analysis of these plots, we have derived the analytical properties of the nanostructured electrodes for the electrochemical sensing of sulfite, such as linear concentration range, sensitivity, detection limit, and reproducibility (see Table S1 in Section 9 of the Supporting Information). Note that the reproducibility data indicate that the building of the different modified electrodes is quite consistent. Furthermore, the sensitivity (slope of the linear range of the calibration curve) of GP-Cu(I) is twice that of GP-Cu(II). Despite the drawbacks concerning the area quantification , as both systems manifest a rather similar porous morphology in terms of roughness, fractality, and nanoparticle size, the higher sensitivity of GP-Cu(I) highlights the relevant role of the copper oxidation state for an enhanced catalytic response.

This major conclusion is further supported by the sensitivity obtained for ED-Cu, which is equal to that of GP-Cu(II) (see Figure 4B). Although they have disparate morphologies (nanorod network and nanoparticulate film, respectively), they do exhibit the same copper oxidation state and, in principle (see above), a similar active surface area. The perfect overlapping of curves *b* and *c* indicates that not only the sensitivity but also the total yield are similar.

### Atomistic Insights into the Sulfite Oxidation Mechanism by DFT

With the main goal of rationalizing the origin of the different catalytic efficiencies of the Cu(I) and Cu(II) catalysts, we follow possible sulfite oxidation reaction paths on both surfaces by DFT-based Gibbs free-energy thermochemical calculations (Computational Details section in the Supporting Information). As the starting point, the balanced master oxidation reaction of interest in aqueous solution is
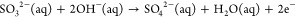
1

We have analyzed, from the theoretical workbench, the energetic viability of different possible mechanisms toward the on-surface oxidation of SO_3_^2–^ into SO_4_^2–^, on both CuO(111) and Cu_2_O(111) surfaces, to understand the role of the oxidation state of Cu (see Figure S11 for a description of the active adsorption sites in both surfaces). We find a viable reaction path, which, for both surfaces, mandatorily involves the previous adsorption of hydroxyl (OH) groups coming from the electrochemical environment on the surface. Within this mechanism, eq 1 translates on-surface into the following elementary intermediate subreactions
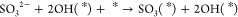
2
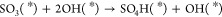
3
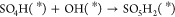
4
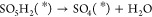
5
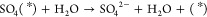
6where “*” and “(*)” refer to available surface-active sites and adsorbed species, respectively.

**Figure 5:**
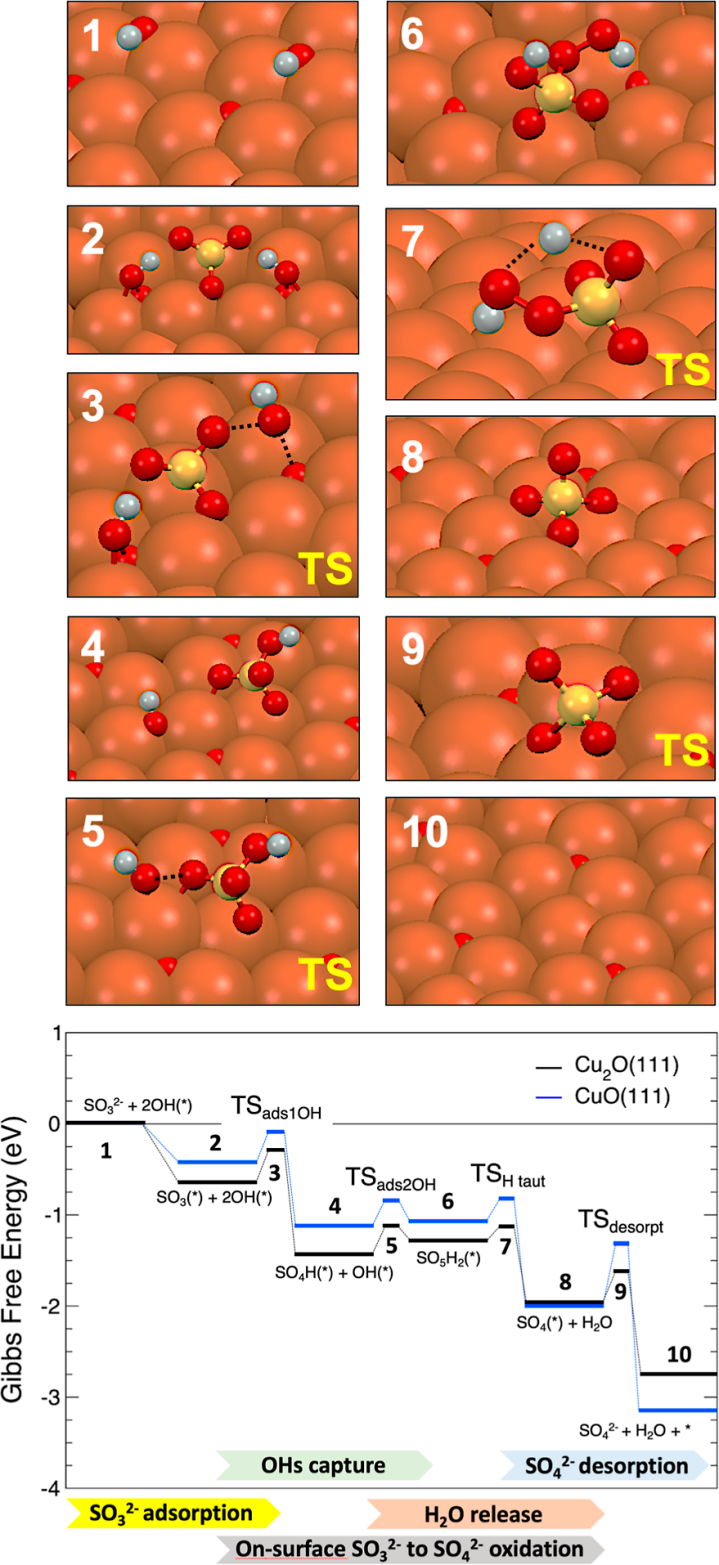
DFT-optimized structures of each intermediate reaction step for the reaction path on the Cu_2_O surface (top) and Gibbs free energy diagram (bottom) for the on-surface SO_3_^2–^ oxidation into SO_4_^2–^ on the Cu_2_O(111) and CuO(111) surfaces at 300 K. White, red, yellow, and orange spheres represent the H, O, S, and Cu atoms, respectively.

The reaction proceeds as follows: in a first step (eq 2), a SO_3_^2–^ anion adsorbs onto a Cu active catalytic center of a CuO/Cu_2_O(111) surface with two previously adsorbed OH groups on top of surface oxygen atoms at a compatible distance of the adsorbed SO_3_^2–^ to initialize the reaction (see Figure 5). This leads to two consecutive captures of the preadsorbed OH groups toward the formation of the adsorbed SO_5_H_2_ species (eqs 3 and 4). Within the adsorbate, a proton migration induces the release of a H_2_O molecule to the electrolyte, leaving an adsorbed SO_4_ (eq 5), which is finally released to the aqueous environment in form of a SO_4_^2–^ anion (eq 6).

To simulate this set of reactions, we have first built two CuO(111) and Cu_2_O(111) canonical surfaces. Our choice of extended infinite slab surfaces was motivated by the substantial size of the experimental copper oxide nanoparticles and nanorods, where quantum boundary effects are not anticipated to impact the catalytic performance. The selection of the most stable (111) face for both Cu_2_O and CuO is grounded in well-established precedents, encompassing both experimental and theoretical studies.^34−36^

Figure 5 shows the computed Gibbs free-energy diagram for the on-surface sulfite SO_3_^2–^ anion oxidative conversion into sulfate SO_4_^2–^ on the Cu_2_O(111) and CuO(111) surfaces at standard conditions of 300 K. The bottom panel shows the DFT-optimized structures of each intermediate reaction step for the reaction path on Cu_2_O surface (see Figure S12 for the corresponding intermediate DFT-optimized structures on the CuO surface).

Initially, the SO_3_^2–^ anion is adsorbed on the copper oxide surfaces on top of a Cu-site active center. In this location, the S atom is at a distance of around 2.2 Å from the Cu, one of the O atoms of the SO_3_(*) bonds to a surface Cu–Cu bridge, and the other two O atoms point up without bonding to the surface (1 → 2). This first adsorption provides net gains of free energy of Δ*G*_1→2_ = −0.67 and −0.45 eV (exothermic) on Cu_2_O and CuO, respectively. From this adsorption configuration, in the sub-reaction 2 → 4, the adsorbed SO_3_(*) captures one OH group preadsorbed on the surface to form the adsorbed SO_4_H(*) species with net gains of free energy of Δ*G*_2→4_ = −0.78 and −0.71 eV (exothermic) and overcoming energy barriers of 0.36 and 0.35 eV on Cu_2_O and CuO (step 3 in Figure 5), respectively. The capture of the OH group to form the SO_4_H(*) weakens the Cu–S bond and leaves the formed adsorbate bonded to the surface by the oxygen located on top of a Cu atom with a bond length of around 1.9 Å.

Subsequently, during sub-reaction 4 → 6, the adsorbed SO_3_H(*) captures an additional OH group preadsorbed on the surface to form the adsorbed SO_5_H_2_(*) species, in this case, with a net loss of free energy of Δ*G*_4→6_ = +0.15 and +0.06 eV (endothermic) for Cu_2_O and CuO, respectively. This reaction is slightly energetically unfavorable due to the increased bond saturation in the adsorbate after the first OH capture. Nonetheless, the computed energy barriers for this subreaction are even lower than for the previous one, with values of 0.19 and 0.23 eV for Cu_2_O and CuO (step 5 in Figure 5), respectively. In this case, the capture of the second preadsorbed OH group still leaves the SO_5_H_2_(*) adsorbate anchored to the surface by the same oxygen on the Cu atom, like in step 4. Note that, at this point, the surface is locally further oxidized, compatible with Cu(III) species.^37^ Thus, from step 6 and in order to favor the release of a water molecule while leaving adsorbed a SO_4_(*), a proton transfer/migration occurs from one of the OH terminal groups of the SO_5_H_2_(*) toward the other OH terminal group to form a terminal OHH. This is released to the aqueous environment as a water molecule in a spontaneous barrierless process (6 → 8). This H-tautomerization subreaction proceeds with significant gain free energy gains of Δ*G*_6→8_ = −0.67 and −0.85 eV (exothermic) and needs to overcome energy barriers of 0.15 and 0.23 eV for Cu_2_O and CuO (step 7 in Figure 5), respectively.

In the final elementary subreaction, the SO_4_(*) adsorbate is released as a SO_4_^2–^ sulfate anion. This reaction is very favorable energetically on both copper oxide surfaces with net free energy gains of Δ*G*_8→10_ = −0.78 and −1.13 eV (exothermic) for Cu_2_O and CuO, respectively. It is interesting to notice that the net gain of free energy is higher for the case of CuO with around 0.35 eV in absolute value. Nonetheless, the computed energy barriers associated with this subreaction are 0.33 and 0.70 eV for Cu_2_O and CuO, respectively, i.e., the barrier is two times lower on the Cu_2_O surface. Thus, despite the net energy gain in the release of SO_4_^2–^ on Cu_2_O, the lower energy barrier implies that the process is favored on the Cu_2_O surfaces. Note that this energy barrier is the limiting step and is the main difference for the reaction on both surfaces as the rest of the energy barriers of the transition states of the whole process are similar for both surfaces.

This finding agrees with the higher experimental efficiency of Cu_2_O nanoparticles toward the SO_3_^2–^ oxidative conversion into sulfate SO_4_^2–^ (see Figure 4). On CuO, the higher energy barrier of 0.70 eV prevents the efficient release of SO_4_^2–^, which slows down the reaction by increasing the residence time of SO_4_^2–^ and “temporarily poisoning” the surface with nonreleased SO_4_ adsorbates.

In step 8, once the SO_4_(*) is formed, this adsorbate accumulates a net electronic charge coming from the Cu_2_O and CuO surfaces of around −1.6 and −1.1 e^–^, respectively. The fact that in Cu_2_O(111), the charge transfer of −1.6 e^–^ is so close to the charge state of −2 of the SO_4_^2–^ sulfate anion dramatically favors the release 8 → 10 reaction over the CuO(111), where the SO_4_(*) is just charged with −1.1 e^–^.

The difference in charge transfer from the surface can be explained by considering the electronic structures of the Cu_2_O(111) and CuO(111) surfaces involved in the calculations as well as the relative positioning of the SO_4_ electronic levels. When the SO_4_(*)/surface interface is formed, the chemical potential of the adsorbate and the Fermi energy of the oxides align due, in this case, to charge transfer from the substrate to the adsorbate.

**Figure 6:**
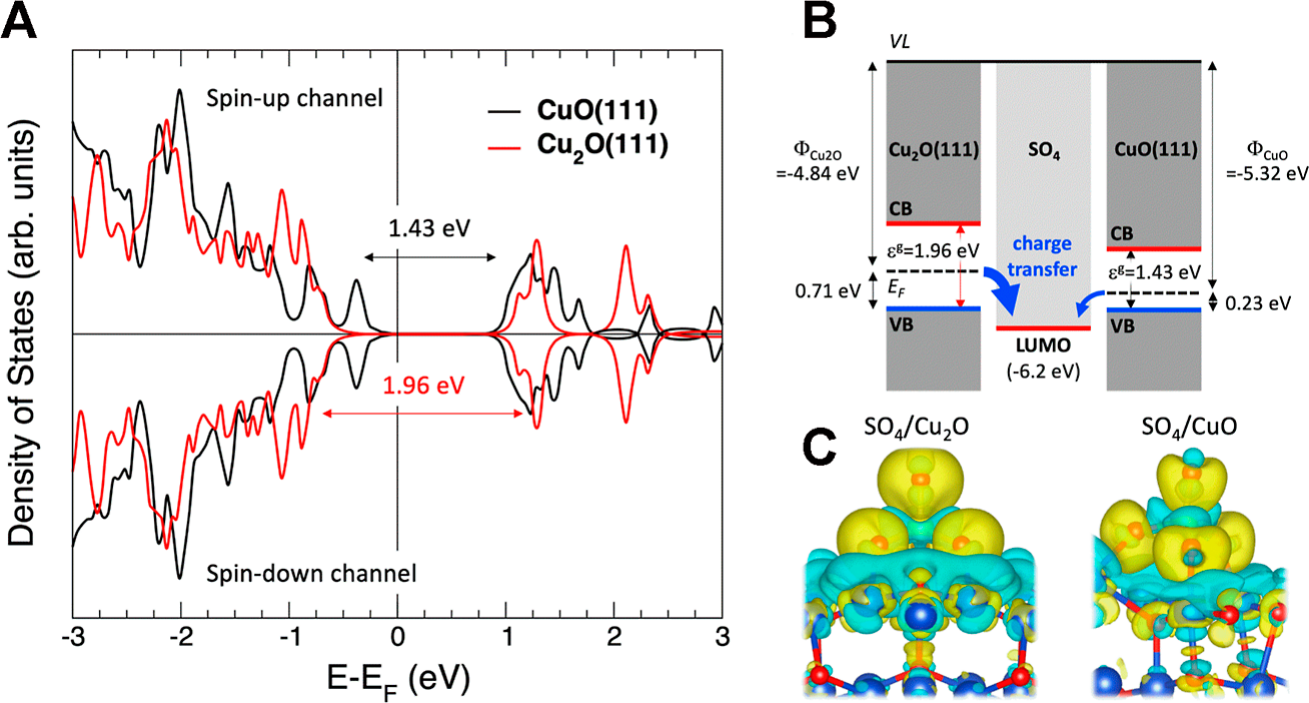
(A) Computed spin-polarized density of states as a function of the energy (in eV), referred to the Fermi energy, for the CuO(111) and the Cu_2_O(111) surfaces at the GGA-U level of theory for an improved electronic description of the d(Cu) states, for which the Hubbard U parameter has been set to 4 eV for the Cu.^38^ (B) Pictorial (and scaled) representation of the electronic levels in both surfaces and in SO_4_ (just LUMO). (C) 3D isosurfaces associated with the difference between the total electron density and the electron densities of the adsorbate and surface counterparts (with the same isovalue of 0.0001 au for a direct comparison) for SO_4_(*) on Cu_2_O(111) and CuO(111). Light blue and yellow isosurfaces represent charge depletion and net charge gain spatial regions, respectively.

In a first step, we have computed the spin-polarized density of states for the CuO(111) and the Cu_2_O(111) surfaces at the GGA-U level of theory for an improved electronic description of the d(Cu) states (Hubbard U parameter set to 4 eV for Cu^38^). These density of states profiles are shown in Figure 6, where it is possible to observe that the electronic structure of both surfaces yields computed gap values of 1.43 and 1.96 eV for CuO(111) and Cu_2_O(111), respectively. These values are comparable to those reported for the CuO and Cu_2_O surfaces on TiO_2_ heterojunctions,^39^ with an excellent agreement with the values obtained in the present study. No difference between channels spin-up and spin-down is observed in Figure 6A.

The difference in the band gaps implies different chemical reactivities of both surfaces, whose origin comes from the different proximities of the valence band to the Fermi energy in both oxides (both conduction bands are essentially located at a similar energy above the Fermi level). Nonetheless, to understand the higher charge transfer from the substrate in Cu_2_O, we have analyzed the energy difference between the Fermi level of both surfaces and the LUMO level of the adsorbate, which provides a measure of the charge transfer needed from the Fermi level to the LUMO toward a common aligned Fermi level in the interface. Figure 6B shows a pictorial (and scaled) representation of the electronic levels of both surfaces and the LUMO of SO_4_. The Fermi energies have been set for the surfaces to their work function values of −4.84 and −5.32 eV^40^ for Cu_2_O and CuO, respectively. The SO_4_ LUMO energy has been determined as its electron affinity by the GAUSSIAN simulation package^41^ at a B3LYP/cc-pVTZ level of theory,^42,43^ yielding a value of −6.2 eV with respect to the vacuum level. In Figure 6B, it is possible to appreciate how the charge transfer from the Fermi level of the Cu_2_O(111) surface to the SO_4_ LUMO has to be higher than in the case of CuO, which explains the difference between −1.6 and −1.1 e^–^. Besides, in order to visualize this more pronounced charge transfer from Cu_2_O, we have depicted in Figure 6C, the 3D isosurfaces associated with the difference between the total electron density and the electron densities of the adsorbate and surface counterparts (with the same isovalue of 0.0001 au for a direct comparison). In this representation, the charge transferred from the surface (light blue), the charge depletion region, and the charge accumulated by the adsorbate (yellow), the net charge gain region, are significantly more pronounced for the case of Cu_2_O, mainly locating, as expected, onto the O atoms of the adsorbed SO_4_. All the above-mentioned points explain the significantly more restrictive limiting reaction step for the case of CuO and the best catalytic performance for this reaction by Cu_2_O.

## Conclusions

We have studied the electrooxidation of sulfite ions to sulfate on copper oxide nanostructured surfaces in an alkaline medium. Films with different stoichiometries and morphologies of Cu-based nanoparticles and CuO-based nanorods were grown on GCEs and examined by using cyclic voltammetry. Our results show that the oxidation state of copper plays a key role in the electrocatalytic response of Cu-based nanostructures. Specifically, Cu(I) displays a better sensitivity and a lower detection limit toward sulfite determination than Cu(II).

Theoretical models suggest that the reaction mechanism involves a tautomerization step on adsorbed OH groups on both Cu(I) and Cu(II) surfaces, with similar energy gains but different charge transfer dynamics, which, in turn, affects the sulfate desorption process. The charge transfer from the surface to the sulfate product differs, leading to a higher desorption barrier on the Cu(II) surface, which is due to the interaction of the sulfate ion with the surface occupied states.

Our results point out the key role played by the copper oxidation state in the catalytic performance of nanostructured films. These findings not only provide a deeper comprehension of the mechanism governing the catalytic behavior of copper oxide nanostructures but also have significant implications for the strategic design and synthesis of new catalysts, particularly for reactions involving sulfur-containing compounds.

## Supporting Information Available

Sample preparation, experimental and theoretical methods, estimation of the active area, TEM images of GP-Cu NPs, fractal analysis of AFM images of GP-Cu(I) and GP-Cu(II) surfaces, emersion experiments with surface morphological and chemistry analysis, electrochemical response toward sulfite and analytical properties of the Cu-modified electrodes, and DFT-optimized CuO(111) and Cu_2_O(111) surfaces as well as of the structures of the reaction steps on the CuO surface (PDF)
