# Supplementary material for: Enhanced Electrocatalysis on Copper Nanostructures: Role of the Oxidation State in Sulfite Oxidation
Source: ACS Catal. 2024 Jul 19;14(15):11522–31. doi: 10.1021/acscatal.3c05897 (PMC11302185; doi:10.1021/acscatal.3c05897)
Supplement: Supplementary file 1 — cs3c05897_si_001.pdf [file cs3c05897_si_001.pdf]

Supplementary materials for

Enhanced Electrocatalysis on Copper Nanostructures: role of the Oxidation State in Sulfite Oxidation

Esperanza Fernández-García[a], Pablo Merino[b], Nerea González-Rodríguez[a], Lidia Martínez[b], María del Pozo[a], Javier Prieto[b], Elías Blanco[a], Gonzalo Santoro[c], Carmen Quintana[a], María Dolores Petit-Domínguez[a], Elena Casero[a], Luis Vázquez[b]*, José I. Martínez[b], José A. Martín-Gago[b]*

[a]Departamento de Química Analítica y Análisis Instrumental. Facultad de Ciencias. c/ Francisco Tomás y Valiente, Nº7. Campus de Excelencia de la Universidad Autónoma de Madrid. 28049 Madrid. Spain

[b] Instituto de Ciencia de Materiales de Madrid ICMM (CSIC). Madrid E-28049. Spain

[c] Instituto de Estructura de la Materia (IEM), CSIC. c/ Serrano 121. 28006 Madrid. Spain

* Corresponding authors: María Dolores Petit-Domínguez (mdolores.petit@uam.es) & Luis Vázquez (lvb@icmm.csic.es)

Table of contents

1. **Methods** **3**

1.1 Synthesis of Cu-based NPs from the gas-phase and deposition on GCE **3**

1.2 Synthesis by electrodeposition of Cu-based nanostructures on GCE **3**

1.3 Electrochemical measurements **6**

1.4 XPS measurements **7**

1.5 TEM measurements **7**

1.6 SEM measurements **8**

1.7 AFM measurements **8**

1.8 Computational details **8**

2. **Estimation of the active area** **11**
3. **TEM images of gas-phase synthetized Cu-based NPs** **12**
4. **Procedure for the fractal analysis of AFM images** **13**
5. **Fractal analysis of the AFM data for GP-Cu(II) and GP-Cu(I)** **15**
6. **Ex-situ emersion experiments: Characterization of the electrode morphologies after the catalytic process** **16**
7. **Ex-situ emersion experiments: Characterization of the electrode surface chemistry after the catalytic process** **18**
8. **Electrochemical response towards sulfite of Cu-modified GCEs** **19**
9. **Analytical properties of the Cu-modified electrodes** **20**
10. **DFT-optimized CuO(111) and Cu_2O(111) surfaces** **21**
11. **DFT-optimized structures of the reaction steps on CuO surface** **22**
12. **References** **23**

1. Methods

1.1. Synthesis of Cu-based NPs from the gas-phase and deposition on GCE

Cu, Cu_2O and CuO nanoparticles (hereafter denoted as GP-Cu(0), GP-Cu(I) and GP-Cu(II), respectively) were fabricated using a scaled-up MICS from Oxford Applied Research Ltd. working in UHV (base pressure $1 \cdot 10^{-9}$ mbar) [1]. The 2” magnetron used in the experiments was loaded with a copper target (99.99% purity). The sputtering gas (Ar, 99.999% purity) flow rate was kept constant for all the experiments at 150 sccm. In addition, oxygen injection during fabrication was carried out through the lateral entrances of the aggregation zone, using extra-pure O_2 (99.999% purity) as explained elsewhere [1]. The O_2 flow rates used for these experiments were: 0, 8 and 21 sccm, keeping an Ar flow rate of 150 sccm. The typical power applied to the magnetron was 45 W. When O_2 is injected, the power typically increases up to 53 W.

The synthesized NPs were deposited on bare glassy carbon electrodes (GCE) of 9 mm inner diameter (ϕ) (Micro to Nano) polished with 1 μm diamond paste (Buehler), rinsed with water and dried with nitrogen. The deposition time on GCE of each sample was adjusted considering the different production rates when O_2 is injected so as to have a total load of 300 ng/cm^2 of nanoparticles, measured with a quartz microbalance, in each sample (20 min for Cu(0), 20 s for Cu(I) and less than 10 s for Cu(II)).

1.2 Synthesis by electrodeposition of Cu-based nanostructures on GCE

Polished GCE were used as working electrodes either with ϕ = 3 mm (Metrohm AG) or ϕ = 9 mm (Micro to Nano). Prior to the electrodeposition process, a cyclic voltammogram was registered between -1.0 and +0.8 V in 0.1 M KCl (Fig. S1 curve a) to assure an adequate surface free of any impurity susceptible to give an electrochemical

signal. At this stage, we have also recorded the cyclic voltammogram of the GCE in a 1.7 mM solution of $CuCl_2$ in 0.1 M KCl (Fig. S1, curve b). As expected, in the anodic sweep two redox peaks appear that correspond to the oxidation from Cu(0) to Cu(I) and from Cu(I) to Cu(II), respectively. In the cathodic sweep, the two inverse processes are observed, the reduction from Cu(II) to Cu(I) and from Cu(I) to Cu(0).

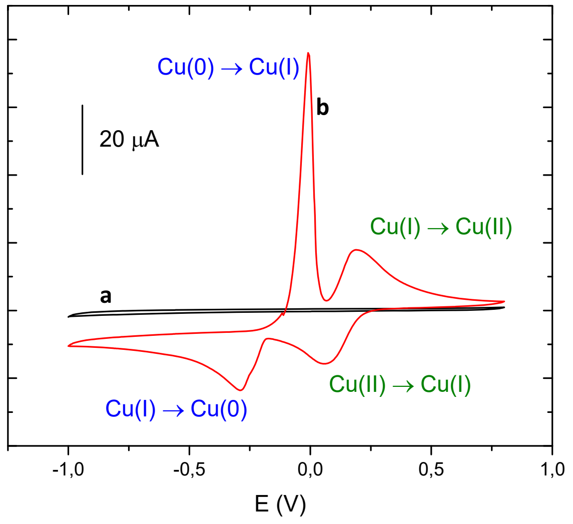

Figure S1. Cyclic voltammograms obtained using a GCE (ϕ = 3 mm) in (a) 0.1 M KCl solution and (b) 1.7 mM $CuCl_2$ in 0.1 M KCl solution. Scan rate 100 mV s^{-1}.

As can be observed in Fig. S1 curve b, a potential value of -0.4 V is adequate to reduce the copper ions on the electrode surface. Accordingly, the Cu electrodeposition was carried out by introducing the GCE in a deaerated 1.7 mM $CuCl_2$ solution in 0.1 M KCl and applying this potential during 180 s. In order to verify that the electrodeposition was successful, the voltammetric response of the electrode was recorded in an electrolyte solution free of copper (0.1 M NaOH). The anodic and cathodic scans of the cyclic voltammogram (Fig. S2A) show the oxidation from Cu(0) to Cu(I) and Cu(II), as well as the reduction from Cu(II) to Cu(I) and Cu(0), occurring through the reactions described below [2].

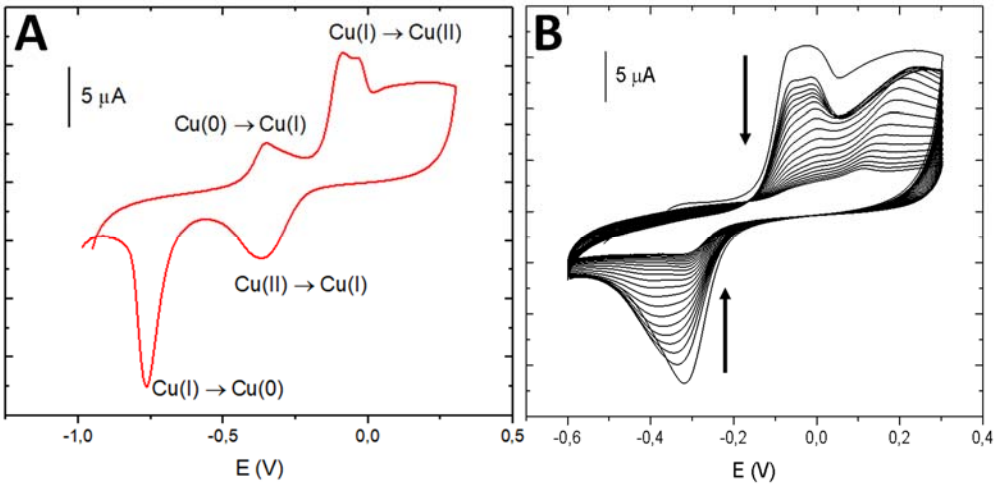

Figure S2. (A) Cyclic voltammogram obtained with the GCE (ϕ = 3 mm) after the electrodeposition process. (B) 20 consecutive scans between -0.6 and +0.3 V obtained with the same electrode. Measurements were performed in 0.1 M NaOH. Scan rate 50 mVs^{-1}.

In Fig. S2A, when advancing in the anodic direction, the oxidation from Cu(0) to Cu(I) occurs in the first place through the following reactions [2]:

$$Cu + OH^- - e^- \rightarrow CuOH$$

(anodic sweep, first peak)

$$2CuOH + OH^- \rightarrow Cu_2O + 2H_2O$$

Then, the oxidation of Cu(I) to Cu(II) occurs, that is, the Cu(OH) and Cu_2O species oxidize to form CuO:

$$CuOH + OH^- - e^- \rightarrow CuO + H_2O$$

(anodic sweep, second and third peaks)

$$Cu_2O + 2OH^- - 2e^- \rightarrow 2CuO + H_2O$$

In the cathodic sweep, the reduction from Cu(II) to Cu(I) appears first:

$$CuO + H_2O + e^- \rightarrow CuOH + OH^-$$

(cathodic sweep, first peak)

$$2CuO + H_2O + 2e^- \rightarrow Cu_2O + 2OH^-$$

And finally, the reduction from Cu(I) to Cu(0):

$$CuOH + e^- \rightarrow Cu + OH^-$$

(cathodic sweep, second peak)

$$Cu_2O + H_2O + 2e^- \rightarrow 2Cu + 2OH^-$$

It is well-known that in order to obtain a stable copper oxide deposit onto an electrode surface is necessary to perform several cycles in an alkaline medium [2, 3]. Accordingly, we applied 20 cycles between -0.6 and +0.3 V. The corresponding cyclic voltammograms are shown in Fig. S2B, in which all the peaks corresponding to the Cu(II)/Cu(I) redox processes decreased as the copper oxide film is formed on the electrode surface. In these conditions, a copper oxide nanowire film forms on GCE, as shown in the corresponding AFM images displayed in the main text. This sample will be referred to as ED-Cu.

1.3 Electrochemical measurements

Electrochemical experiments were done with an Ecochemie Autolab PGSTAT302N system employing either the bare or Cu-modified GCE described above as working electrode. To complete the three-electrode configuration electrochemical cell, Ag/AgCl (3.0 M KCl) and a platinum wire were used as reference and auxiliary electrodes, respectively. Solutions were deaerated with nitrogen gas before use, and the gas flow was kept over the solutions during experiments. Solutions were prepared just

prior to use with purified water obtained from a Millipore Milli-Q-System. All reagents employed in this work were purchased from Sigma–Aldrich.

The electrochemical response of the Cu-modified electrodes towards sulfite was evaluated by cyclic voltammetry in NaOH 0.01 M using the following potential sweep: +0.25 V → +0.85 → +0.25 V at 50 mVs^{-1}, in the absence and in the presence of sulfite. Calibration curves were obtained by averaging the measurements on five different modified electrodes for each system.

1.4 XPS measurements

The GP samples were analyzed in-situ by X-ray photoelectron spectroscopy (XPS) with a PHOIBOS 100 1D electron/ion analyzer with a one-dimensional delay-line detector. X-ray photons of 1486.6 eV (Al Kα emission line) were supplied by a water-cooled commercial X-ray gun XR 50 M (Specs) coupled to a Focus 500 X-ray monochromator (Specs). The absolute binding energies (BE) of the photoelectron spectra were determined by referencing the C 1s at 284.8 eV. For this characterization, the analyzed films were thinner than those studied electrochemically and by AFM. ED-Cu samples were analyzed ex-situ with the same equipment.

1.5 TEM measurements

The size of the NPs was characterized by ex-situ transmission electron microscopy (TEM) using an amorphous carbon-coated TEM grid as substrate. TEM micrographs were recorded using a TEM/STEM (JEOL 2100F) microscope operated at 200 kV. For this characterization, short deposition times directly on the TEM grid were employed in order to achieve isolated nanoparticles (i.e., 10 min for GP-Cu(0), 1 s for GP-Cu(I) and less than 1 s for GP-Cu(II)).

1.6 SEM measurements

Ex-situ Scanning Electron Microscopy (SEM) was employed to characterize the Cu films morphology before and after the electrocatalytic process using a NOVA NANOSEM 230 equipment (© FEI) operating with a low-voltage and high-contrast detector (vCD).

1.7 AFM measurements

The morphological characterization using atomic force microscopy (AFM) measurements were performed with two systems, namely Nanoscope IIIa (Veeco) and Picoplus 5500 (Agilent). Silicon cantilevers (Bruker) with nominal force constant of 40 N/m and tip radius of 8 nm were employed. The AFM data were acquired ex-situ, under ambient conditions, and using the dynamic mode.

1.8 Computational details

We have carried out a large battery of Density Functional Theory (DFT)-based calculations, adequately complemented with the thermochemical Gibbs free energy formalism, with the main goal of elucidating a viable reaction mechanism to explain the oxidation of SO_3^{2-} into SO_4^{2-} on CuO(111) and Cu_2O(111) surfaces. As a proof-of-concept, in order to mimic the effective surface of the experimentally synthesized CuO and Cu_2O nanoparticles and nanorods with sizes of several nm, we have theoretically studied this reaction on extended 2D infinite surface slabs, with a perfectly balanced stoichiometry as a reasonable approximation to get results directly comparable with the experimental evidence. For this purpose, we have used the DFT and Gibbs free-energy

thermochemistry formalisms as implemented in the plane-wave atomistic simulation package CASTEP [4]. The electronic exchange-correlation has been accounted by the GGA-PBE functional [5], and the ion-electron interaction within each atom-type involved in the systems (H, O, S and Cu) has been modelled by ultrasoft pseudopotentials [6]. Dispersive forces have been included in the calculations by the Tkatchenko-Scheffler van der Waals correction scheme [7], based on a scaling approach that yields in situ atomic polarizabilities (α), dispersion coefficients (C_6), and vdW radii (R_{vdW}) that reflect the local electronic environment. Brillouin zones in all the interfacial model systems has been sampled by optimal Monkhorst-Pack [4×4×1] *k*-point grids [8].

One-electron wave-functions were expanded in a basis of plane waves of 500 eV cutoff for the kinetic energy, and atomic relaxations were performed until the maximum force acting on any atom was below 0.02 eV $Å^{-1}$. The CuO(111) and Cu_2O(111) substrates were modeled as infinite 2D periodic slabs with four physical layers (see Fig. S11), keeping fixed the two bottommost ones during the geometrical optimizations. Systems in neighboring cells along the perpendicular-to-the-surface direction were separated by at least a 20 Å-thick vacuum region to avoid the interaction between two adjacent slabs.

Zero-point energy, temperature and entropy influence have been included into the reaction mechanism by the Gibbs free energy formalism by computing free energies on the basis of the electronic DFT energies at a temperature of 300 K and a standard pressure of 1 atm by the finite displacement method calculation of vibrational frequencies. Gibbs free energies were computed by the following expression:

$$G = H - TS = E_{DFT} + E_{ZPE} + \int_0^T C_v dT - TS, \qquad \text{(eq. S1)}$$

where E_{DFT} is the DFT-optimized total energy at 0 K, E_{ZPE} is the zero-point energy correction, C_v is the heat capacity, T is the reaction temperature and S is the entropy, which includes the translational, rotational and vibrational contributions. We have applied

the linear synchronous transit/quadratic synchronous transit (LST/QST) method to search for the transition states between reactants and products for all elementary reactions, characterized by an only imaginary frequency.

2. Estimation of the active surface area

The determination of the electrochemically active surface area can be performed according to different electrochemical methods. In the particular case of electrodes modified with nanomaterials there is not a single solution, since the problem becomes complex, depending not only on the material but also on its size. Since most of the methods typically employed in the literature are based on assumptions corresponding to macroscopic electrodes, their applicability to nanostructured electrodes lacks accurate validation [9]. In our case, we have applied the most employed method based on the Randles- Sevcik equation. Due to the problems mentioned above, the use of this equation to estimate the real surface area for the electrodes modified with nanomaterials has to be considered with care [10-12]. Furthermore, a key choice in these measurements is the selection of an adequate redox probe. Accordingly, we have used an outer-sphere probe namely, $Ru(NH_3)_6^{2+/3+}$ since it is only influenced by the electronic structure of the electrode surface [10]. Thus, we have recorded cyclic voltammograms at different scan rates for GP-Cu(I), GP-Cu(II), and ED-Cu GCE in a KCl 1M solution containing 10 mM $Ru(NH_3)_6^{2+/3+}$. The electrochemical surface area was obtained from the slope of the Ip vs $v^{1/2}$ plots according to the Randles- Sevcik equation:

$$Ip_a = (2.69\times10^5)\ n^{3/2}\ A\ D^{½}\ v^{½}\ C, \qquad \text{(eq. S2)}$$

where Ip_a is the anodic peak current, n the number of electrons, A the electrochemical surface area, D the $Ru(NH_3)_6^{2+/3+}$ diffusion coefficient (7.9 10^{-6} cm^2 s^{-1} at 22ºC, [13]), v the scan rate, and C the concentration of the $Ru(NH_3)_6^{2+/3+}$ redox probe.

3. TEM images of gas-phase synthetized Cu-based NPs

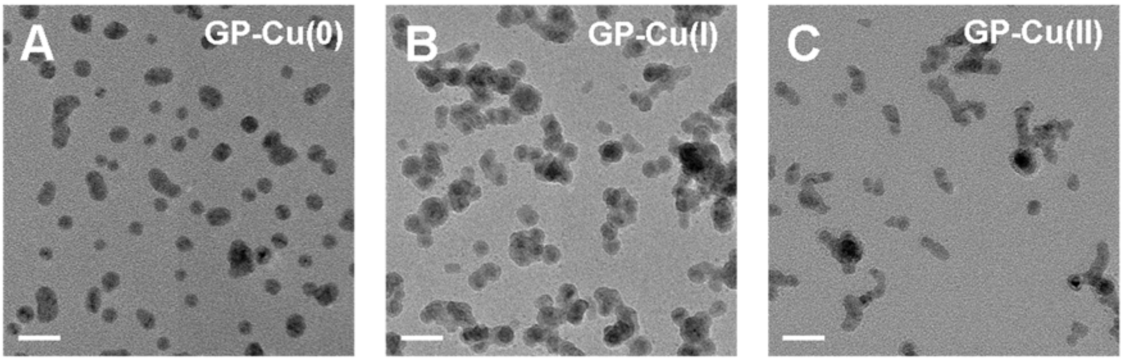

Figure S3. TEM images of **(A)** GP-Cu(0), **(B)** GP-Cu(I) and **(C)** GP-Cu(II). The scale bars correspond to 20 nm.

4. Procedure for the fractal analysis of AFM images

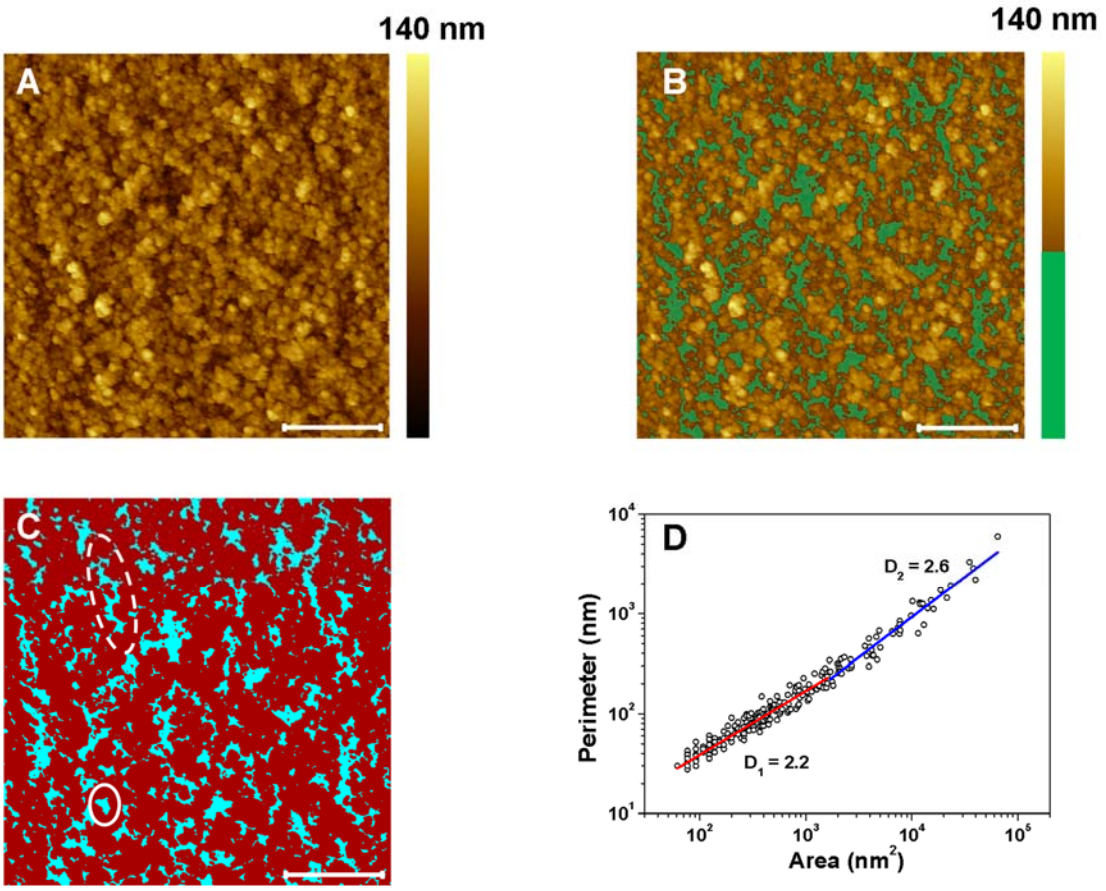

Figure S4. Scheme of the procedure for the fractal analysis of an AFM image of the GP-Cu(I) sample. **(A)** Original image. **(B)** The same image that has been filled up to a given height level threshold with green color, thus defining a network of "islands". **(C)** Resulting binary image where all the islands have zero height (blue zones) and those points above the threshold have 1 height (red zones). The scale bars correspond to 500 nm. **(D)** Logarithmic plot of the perimeter of the island vs. its corresponding area for all island depicted in (C) that do not touch any image's edge.

Fig. S4 depicts the process followed to obtain the fractal behavior of the AFM images [14]. One image of the GP-Cu(I) sample is chosen to be analyzed (Fig. S4A). Then, those points with heights below a threshold level are marked (filled) with green color (Fig. S4B). Afterwards, this image is converted in a binary image where those points below the threshold are given a zero value (blue) whereas those with higher values are given the value 1 (red) (Fig. S4C). This image, then, defines a network of islands (blue). For each island that does not touch any image's edge it is calculated its perimeter and

area. The threshold level is changed. For each value, a new set of islands is generated, as depicted in Fig. S5.

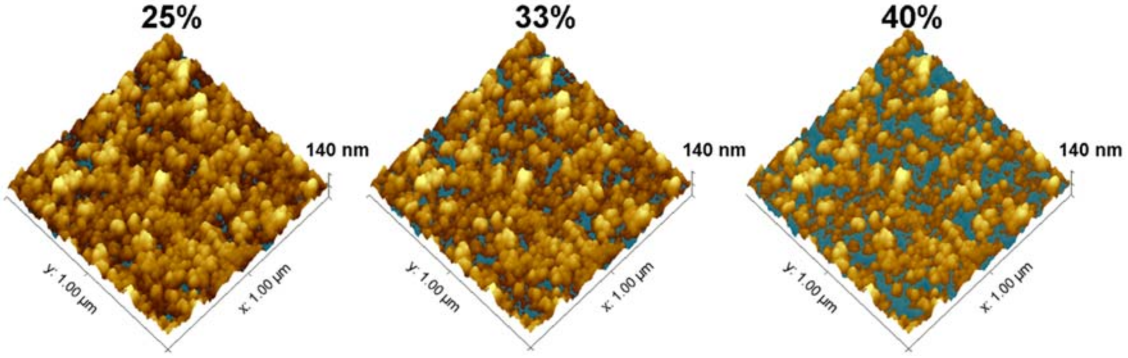

Figure S5: Three-dimensional AFM images of the GP-Cu(I) sample showing the islands defined at different threshold levels of 25% (left), 33% (center) and 40% (right) of the maximum height. For each threshold condition, a different set of islands is defined whose area/perimeter values are included in the plot in Figure S4D.

The perimeter-area data of all the islands generated by changing the threshold levels from the different AFM images are plotted in a logarithmic graph of the perimeter vs. area (Fig. S4D). The ensemble of data usually defines two linear regions with different slopes, from which the corresponding fractal dimension, denoted as D, can be extracted. Note how the small islands (i.e. small area values) define a low D value as they usually have a Euclidean shape without intricate perimeter as such marked within a circle in Fig. S4C. On the contrary, the islands with large area values usually have jagged and intricate perimeters as such marked by the dashed ellipse in Fig. S4C, thus yielding higher D values. Note that the "zero" islands would correspond to pore structures defined at different height levels. Thus, a D value clearly higher than 2 (the Euclidean value) would indicate that the pores have long and jagged perimeters and, thus, a higher number of active sites. The errors involved in the D determination from the slopes of the corresponding plots are less than 5% of the obtained values.

5. Fractal analysis of the AFM data for GP-Cu(II) and GP-Cu(I)

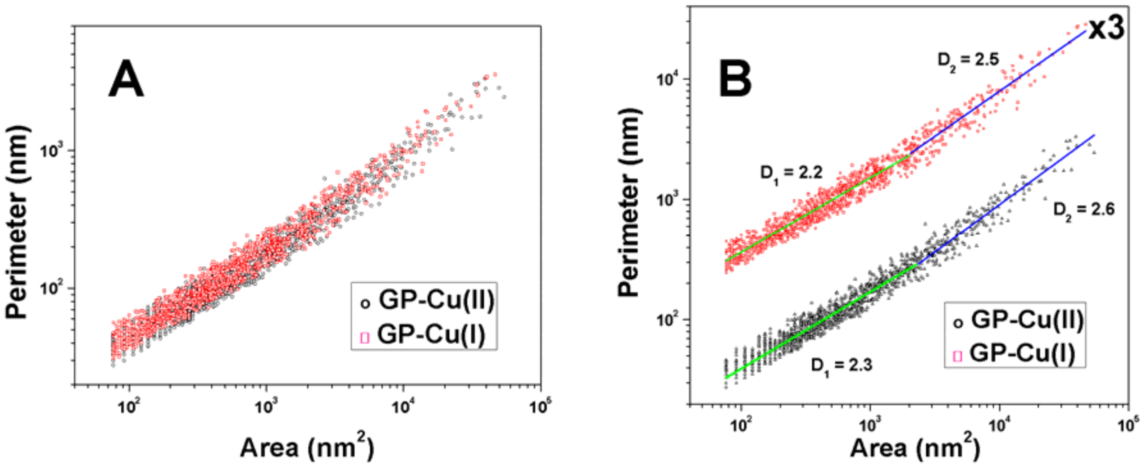

Figure S6. (A) Perimeter vs. Area double logarithmic plots obtained from the AFM images of the (black circles) GP-Cu(II), and (red squares) GP-Cu(I). (B) Same data as on (A) but the Cu(I) y-data are multiplied by 3 for clarity sake. The corresponding linear fittings are indicated by the solid lines, as well as the respective D values for the small (red line) and large (blue) area regions.

Fig. S6A shows the perimeter (P) vs. Area (A) plots obtained after analyzing several images of the GP-Cu(I) and GP-Cu(II) systems. From the overlap of the data for both systems, i.e., similar area/perimeter length ratios, it is clear that they display a similar behavior. In order to better appreciate this similarity, in figure S6B are plotted the same data but, in this case, the y-values of the Cu(I) system are multiplied by 3. Both systems present two regions, with low and high D values, separated by a crossover. For small area values the fractal dimension is close to the Euclidean value of D = 2. These values correspond to the small islands as such marked with a circle in Fig. S4C for which the perimeter profile is rather smooth (i.e, $P \propto A^{1/2}$). In contrast, for larger area values the fractal dimension increases above 2.5, which is associated to the larger structures, as such marked with a dashed ellipse in Fig. S4C, where the perimeter is rather jagged and intricate. The crossover area between both regions (i.e., low and high D values) is close to 2000 nm^2 in both cases. Thus, in terms of the pore morphology and distribution both films are similar [15].

6. **Ex-situ emersion experiments: Characterization of the electrode morphologies after the catalytic process.**

We have studied whether the electrodes after the electrocatalytic process undergo significate changes in their surface morphology.

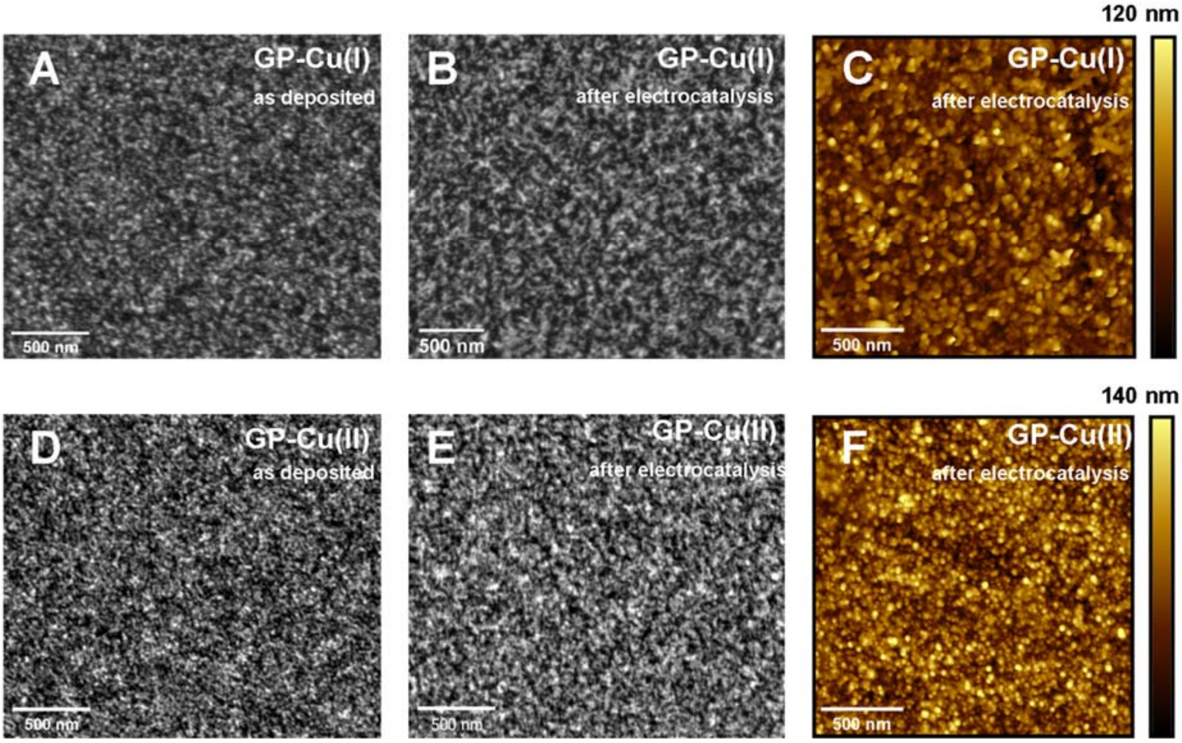

Figure S7. SEM morphologies for the as-grown GP-Cu(I), (A) top left, and GP-Cu(II), (D) bottom left, films. In the middle column the corresponding SEM images after the electrocatalysis are displayed , (B) for GP-Cu(I) and (E) for GP-Cu(II). Finally, Figures (C) and (F) show characteristic AFM images of the GP-Cu(I) and GP-Cu(II) electrodes, respectively, after the electrocatalysis.

Figure S7 shows SEM images of GP-Cu(I) and GP-Cu(II) electrodes before (left column) and after (middle column) the electrocatalytic process. The morphologies are rather similar since they correspond to a compact film formed by nanoparticles. In the right column are also displayed AFM images of both electrode surfaces after the catalytic process. The nanoparticles are visible in both cases. Removal of particles during the electrochemical or the last washing processes can be discarded. Notwithstanding the washing procedure, scarce features can still be observed on the surface that can come

from byproducts of the reaction. These are more evident in the phase-contrast images (not shown).

A similar study was performed with the ED-Cu films. Figures S8A and S8C show the nanorod morphology imaged by SEM and AFM, respectively. In this case, a sort of branched star-like morphology is developed, which is not altered by the catalytic process since the nanorods are not changed nor disassembled (Figures S8B and S8D).

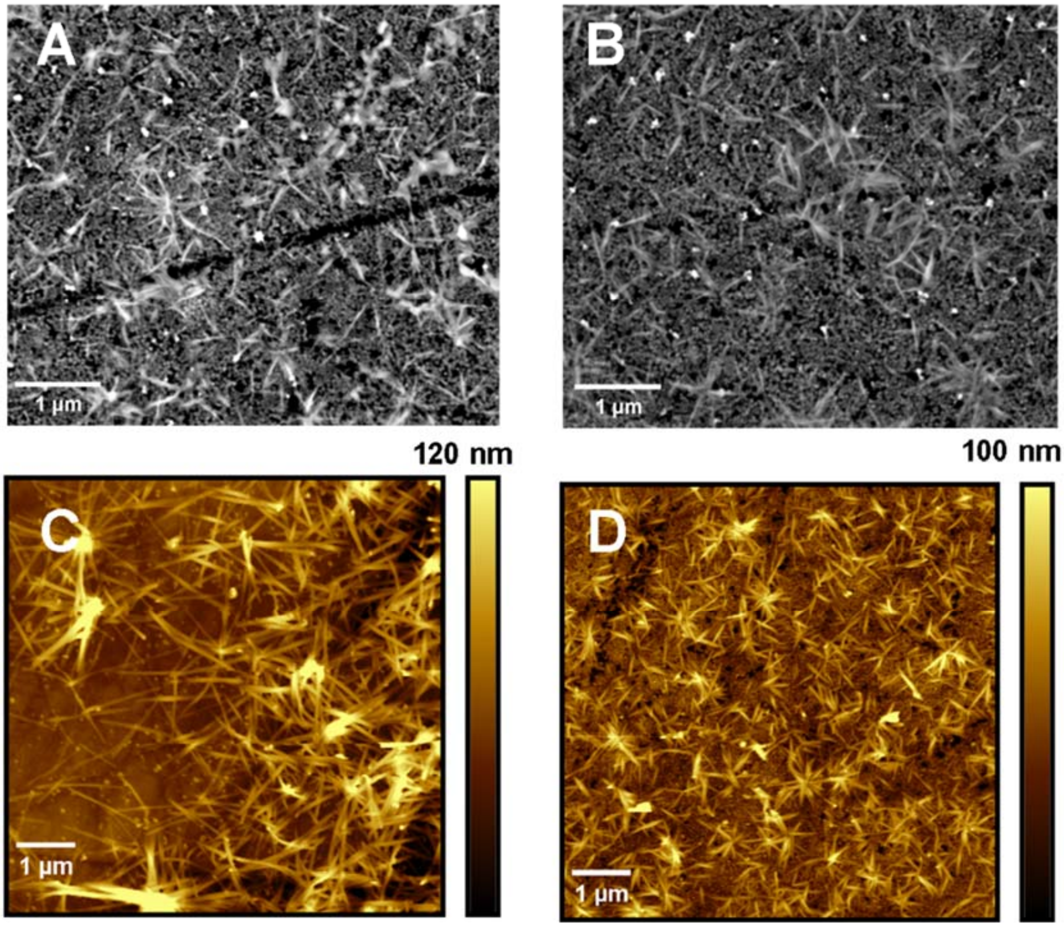

Figure S8. SEM (A, B) and AFM (C, D) morphologies for the ED-Cu films before (A, C, left column) and after (B, D, right column) the electrocatalytic process.

7. Ex-situ emersion experiments: Characterization of the electrode surface chemistry after the catalytic process.

Likewise, we have studied the surface chemistry of the electrodes after been subjected to the electrocatalytic process.

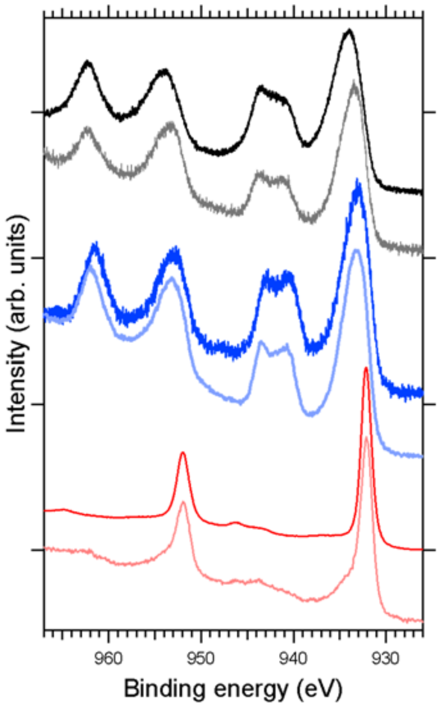

Figure S9. Comparison of XPS Cu 2p core level spectra of ED-Cu (top, black), GP-Cu(II) (middle, blue) and GP-Cu(I) (bottom, red) before (darker curves) and after (lighter curves) electrocatalysis.

Figure S9 presents a comparison of the Cu 2p core level spectra of the samples before and after the electrocatalysis. In the case of the ED-Cu sample (black), there is a small shift of the main peak towards lower BE. This fact, together with the shape of the shake-up satellite, indicates that the presence of hydroxides is not as clear as before the electrocatalysis and the sample has a larger proportion of CuO. The GP-Cu(II) sample (blue) clearly maintains the CuO structure after the electrocatalysis, while the GP-Cu(I) (red) fundamentally keeps the Cu(I) stoichiometry, and only a small shoulder at higher BE (933-935 region) and certain structure between the $Cu2p_{3/2}$ and $Cu2p_{1/2}$ indicate a slight oxidation to Cu(II).

8. Electrochemical response towards sulfite of Cu-modified GCEs

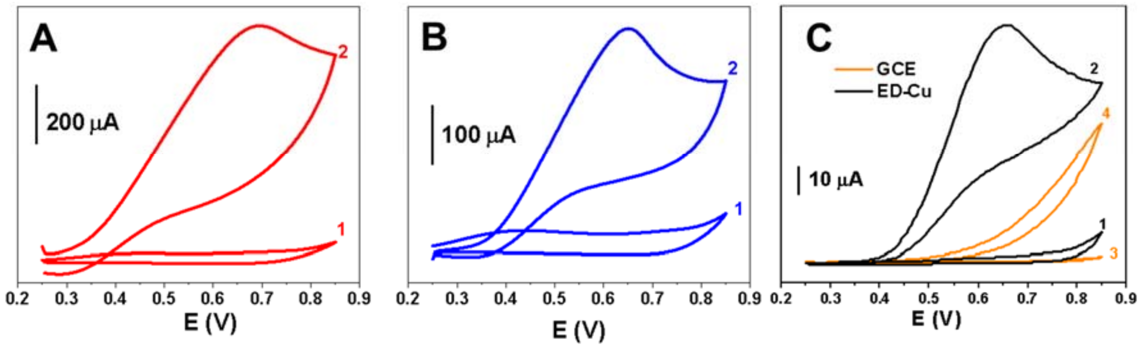

Figure S10. Cyclic voltammograms obtained in 0.01M NaOH in the absence (curves labelled 1) and in the presence of 10 mM Na_2SO_3 (curves labelled 2) for: (A) GP-Cu(I), (B) GP-Cu(II), and (C) ED-Cu modified electrodes. The curves 3 and 4 in C stand for the unmodified GCE in the absence and presence of 10 mM Na_2SO_3, respectively. Scan rate 50 mVs^{-1}. GCE ϕ = 10 mm (A, B) and ϕ = 3 mm (C).

We have evaluated the effect of the GCE modification with Cu nanostructures on the electrochemical response towards sulfite. Figure S10A displays the response of the GP-Cu(I)-modified GCE both in the absence (curve 1) and in the presence (curve 2) of sulfite. The same ensemble of data is shown for the GP-Cu(II)-modified GCE (B) and for the ED-Cu electrode (C). It is clear that, for the three systems, the nanostructured copper film on the electrode, under the presence of sulfite, leads to a well-defined anodic peak at about 0.65 V. Furthermore, Figure S10C also displays the response of the unmodified electrode GCE (orange lines) both in absence (curve 3) and in presence (curve 4) of sulfite.

9. Analytical properties of the Cu-modified electrodes

Table S1. Analytical properties of the Cu-based modified electrodes.

**Sensor**	**ED-Cu**	**GP-Cu**		
		**GP-Cu(I)**	**GP-Cu(II)**	**GP-Cu(0)**
**Linear concentration range (mM)**	0.16-25	0.12-20	0.16-30	0.68-47
**Sensitivity (μA mM^{-1})**	14.0	31.5	14.2	11.3
**Detection limit (mM)**	0.049	0.034	0.046	0.204
**Reproducibility (R.S.D. %, C=10 mM, N=5)**	8.3	7.0	5.1	5.7

From the analysis of the calibration curves displayed in Fig. 4B, the analytical properties of the modified GCEs, such as linear concentration range, sensitivity, detection limit and reproducibility can be extracted (Table S1). The sensitivity was obtained from the slope of the linear range of the calibration curve. The value given in the table is the average of three obtained calibration lines. The detection limit was calculated as the sulfite concentration that provides an equal response to the blank signal plus 3 times its standard deviation (n = 5). Reproducibility was evaluated from the electrochemical response obtained using five different sensors, for a 10 mM concentration of sulfite.

10. DFT-optimized CuO(111) and Cu_2O(111) surfaces

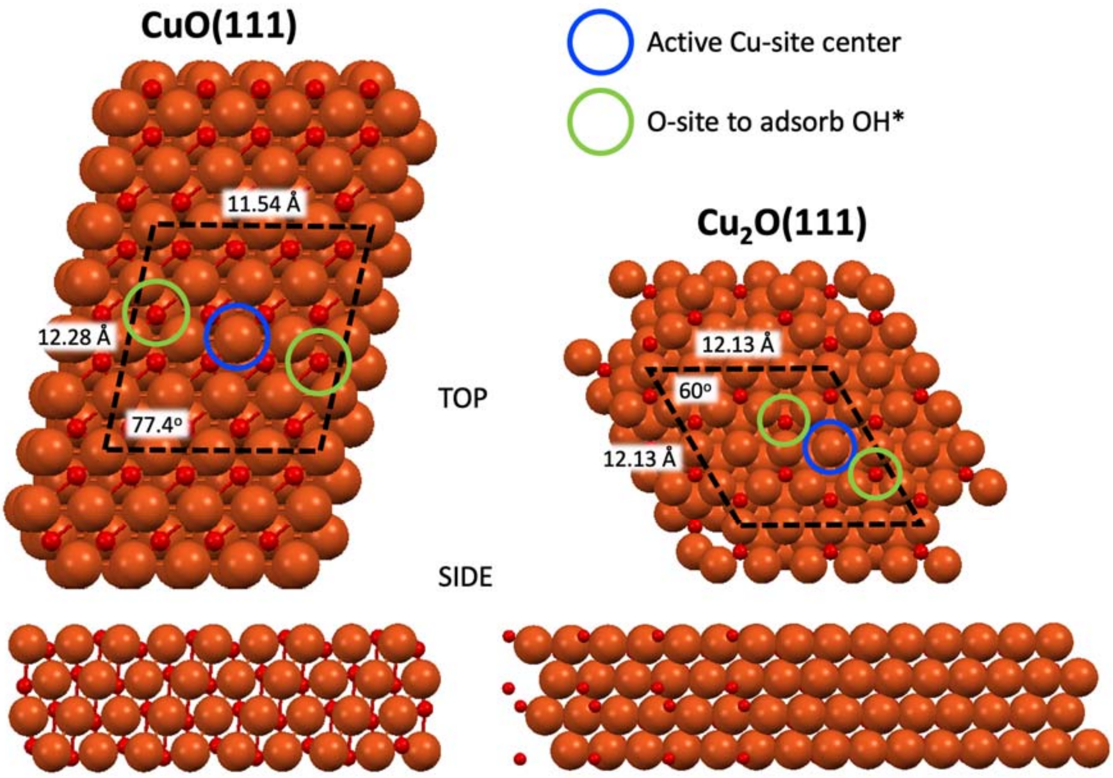

Figure S11. Top and side views of the DFT-optimized CuO (111) (left) and Cu_2O (111) (right) surfaces used across the study. It is indicated for both surfaces the unit cell used in the calculations, represented by a black dashed-line rhomboid, as well as the active Cu-site catalytic center and the most favorable O adsorption sites to anchor OH groups at favorable distances to initialize the reaction.

11. DFT-optimized structures of the reaction steps on CuO surface

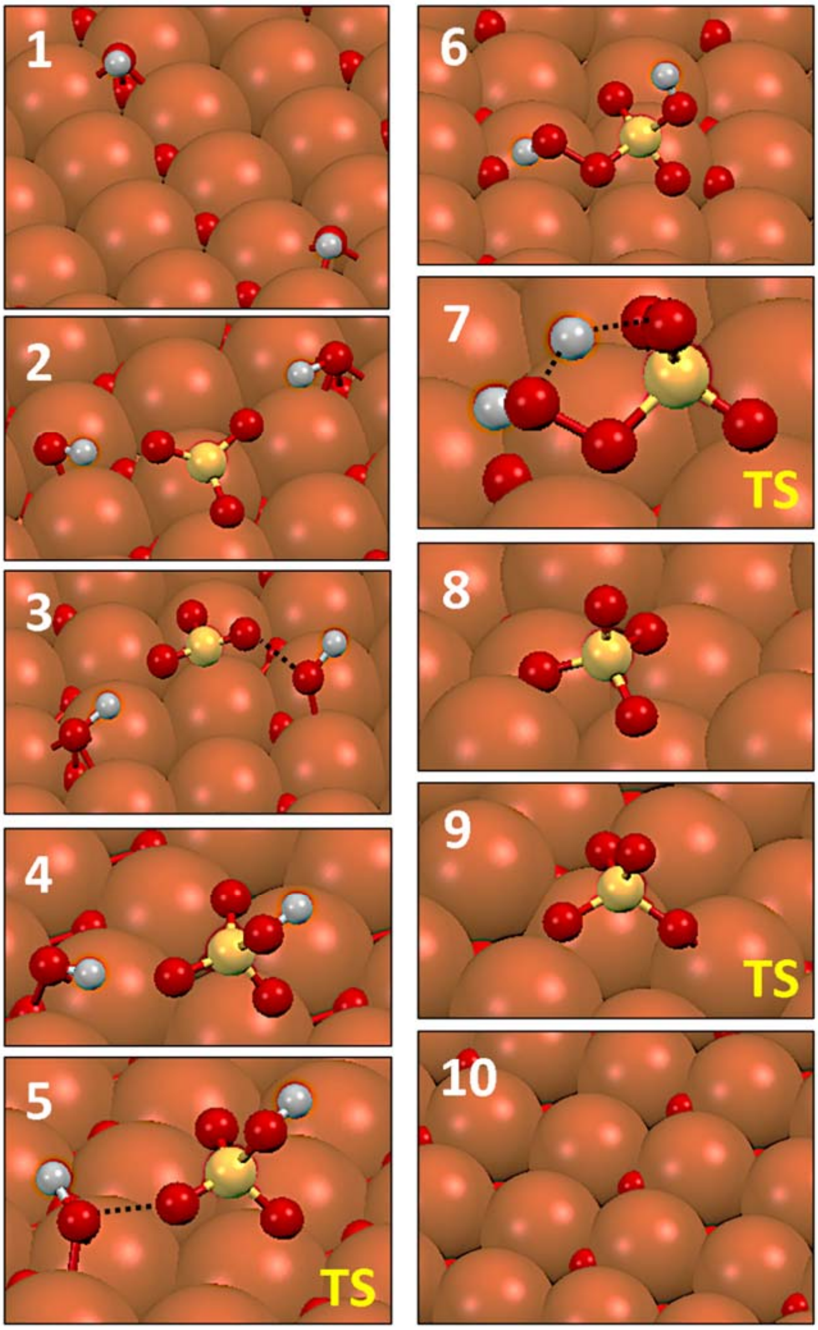

Fig. S12. DFT-optimized structures of each intermediate reaction step (for the reaction path on CuO surface) for the on-surface SO_3^{2-} oxidation into SO_4^{2-} on the CuO(111) surface at 300 K and 1 atm. White, red, yellow and orange spheres represent the H, O, S and Cu atoms, respectively.

12. References

1. Martínez L.; Lauwaet K.; Santoro G.; Sobrado J.M.; Peláez R.J.; Herrero V.J.; Tanarro I.; Ellis G.J.; Cernicharo J.; Joblin C.; Huttel Y.; Martín-Gago J.A. Precisely controlled fabrication, manipulation and in-situ analysis of Cu based nanoparticles. *Sci. Rep.* **2018**, *8*, 7250.
2. Wen-Zhi L.; You-Qin, L. Preparation of nano-copper oxide modified glassy carbon electrode by a novel film plating/potential cycling method and its characterization. *Sens. Actuators B Chem.* **2009**, *141*, 147–153.
3. Fan H.H.; Weng W.L.,; Lee C.Y.; Liao C.N. Electrochemical cycling-induced spiky Cu_xO/Cu nanowire array for glucose sensing. *ACS Omega* **2019**, *4*, 12222−12229.
4. S. J. Clark S.J.; Segall M.D.; Pickard C.J.; Hasnip P.J.; Probert M.J.; Refson K.; Payne M.C. First principles methods using CASTEP. *Zeitschrift für Kristallographie* **2005**, *220*, 567-570.
5. Perdew J.J.P.; Burke K.; Ernzerhof M. Generalized Gradient Approximation Made Simple. *Phys. Rev. Lett.* **1996**, *77*, 3865.
6. Vanderbilt D. Soft self-consistent pseudopotentials in a generalized eigenvalue formalism. *Phys. Rev. B* **1990**, *41*, 7892-7895.
7. Tkatchenko A.; Scheffler M. Accurate Molecular Van Der Waals Interactions from Ground-State Electron Density and Free-Atom Reference Data. *Phys. Rev. Lett.* **2009**, *102*, 073005.
8. Monkhorst, H. J., Pack, J. D. Special Points for Brillouin-Zone Integrations. *Phys. Rev. B.* **1976**, *13*, 5188-5192.
9. Cignoni P.; Hosseini, P.; Kaiser, C.; Trost, O.; Nettler, D-R.; Trzebiatowski, L.; Tschulik, K. Validating Electrochemical Active Surface Area Determination of Nanostructured Electrodes: Surface Oxide Reduction on AuPd Nanoparticles. *J. Electrochem. Soc.* **2023**, *170*, 116505.
10. García-Miranda Ferrari, A.; Foster, C.W.; Kelly, P.J.; Brownson, D.A.C.; Banks, C.E. Determination of the Electrochemical Area of Screen-Printed Electrochemical Sensing Platforms. *Biosensors* **2018**, *8*, 53.
11. Paixao, T.R.L.C. Measuring Electrochemical Surface Area of Nanomaterials versus the Randles-Ševčík Equation. *ChemElectroChem* **2020**, *7*, 3414.
12. Chiticaru, E.A.; Damian, C.M.; Pilan, L.; Ionita, M. Label-Free DNA Biosensor Based on Reduced Graphene Oxide and Gold Nanoparticles. *Biosensors* **2023**, *13*, 797.
13. Beriet, C.; Pletcher D. A further microelectrode study of the influence of electrolyte concentration on the kinetics of redox couples. *J. Electroanal. Chem.* **1994**, 375, 213-218.
14. Herrasti P.; Ocón P.; Salvarezza R.C.; Vara J.M.; Vázquez L.; Arvia A.J. A comparative study of electrodeposited and vapour deposited gold films. Fractal surface characterization through scanning tunneling microscopy. *Electrochim. Acta* **1992**, *37*, 2209-2214.

15. García-Ayuso G.; Salvarezza R.; Martínez-Duart J. M.; Sánchez O.; Vázquez L. Effect of Surface Fractality on the Permeability of Transparent Gas Barrier Coating. Adv. Mater. 1997, 9, 654-658.
